# Genome-wide assessment of genetic risk for systemic lupus erythematosus and disease severity

**DOI:** 10.1093/hmg/ddaa030

**Published:** 2020-02-20

**Authors:** Lingyan Chen, Yong-Fei Wang, Lu Liu, Adrianna Bielowka, Rahell Ahmed, Huoru Zhang, Phil Tombleson, Amy L Roberts, Christopher A Odhams, Deborah S Cunninghame Graham, Xuejun Zhang, Wanling Yang, Timothy J Vyse, David L Morris

**Affiliations:** 1 Department of Medical and Molecular Genetics, King’s College London, London, UK; 2 MRC/BHF Cardiovascular Epidemiology Unit, University of Cambridge, Cambridge, UK; 3 Department of Paediatrics and Adolescent Medicine, LKS Faculty of Medicine, The University of Hong Kong, Hong Kong; 4 Department of Dermatology, NO. 1 Hospital, Anhui Medical University, Hefei, Anhui, China; 5 Key Laboratory of Dermatology, Ministry of Education, Anhui Medical University, Hefei, Anhui, China; 6 Department of Dermatology, Huashan Hospital of Fudan University, Shanghai, China; 7 Department of Twin Research and Genetic Epidemiology, King’s College London, London, UK

## Abstract

Using three European and two Chinese genome-wide association studies (GWAS), we investigated the performance of genetic risk scores (GRSs) for predicting the susceptibility and severity of systemic lupus erythematosus (SLE), using renal disease as a proxy for severity. We used four GWASs to test the performance of GRS both cross validating within the European population and between European and Chinese populations. The performance of GRS in SLE risk prediction was evaluated by receiver operating characteristic (ROC) curves. We then analyzed the polygenic nature of SLE statistically. We also partitioned patients according to their age-of-onset and evaluated the predictability of GRS in disease severity in each age group. We found consistently that the best GRS in the prediction of SLE used SNPs associated at the level of *P* < 1e−05 in all GWAS data sets and that SNPs with *P*-values above 0.2 were inflated for SLE true positive signals. The GRS results in an area under the ROC curve ranging between 0.64 and 0.72, within European and between the European and Chinese populations. We further showed a significant positive correlation between a GRS and renal disease in two independent European GWAS (*P*_**cohort1**_ = 2.44e−08; *P*_**cohort2**_ = 0.00205) and a significant negative correlation with age of SLE onset (*P*_**cohort1**_ = 1.76e−12; *P*_**cohort2**_ = 0.00384). We found that the GRS performed better in the prediction of renal disease in the ‘later onset’ compared with the ‘earlier onset’ group. The GRS predicts SLE in both European and Chinese populations and correlates with poorer prognostic factors: young age-of-onset and lupus nephritis.

## Introduction

Systemic lupus erythematosus (SLE [OMIM: 601744]) is a chronic inflammatory autoimmune disease characterized by a wide spectrum of signs and symptoms varying among affected individuals and can involve many organs and systems including the skin, joints, kidneys, lungs, central nervous system and hematopoietic system (1). A recent report underscores that SLE is among the leading causes of death in young females, particular females among ages 15–24 years, in which SLE ranked 10th in the leading causes of death in all populations and fifth for African American and Hispanic females (2). Lupus nephritis (LN) is the most common cause of morbidity and mortality. Patients with kidney disease are likely to have more severe clinical outcomes and a shorter lifespan. About 30–60% of adults and up to 70% of children with SLE have renal disease, characterized by the glomerular deposition of immune complexes and an ensuring inflammatory response (3). Genetic ancestry influences the incidence and prevalence of SLE and kidney involvement, being more frequent in Hispanics, Africans and Asians than in European (4–7). Currently, kidney disease in SLE is diagnosed by the use of light microscopy, which drives therapeutic decision-making. However, not all patients will respond to therapy, indicating that additional information focusing on the mechanism of tissue injury is required. Moreover, early detection of kidney involvement in SLE is important because early treatment can be applied to reduce the accumulation of renal disability.

Although the exact etiology of lupus is not fully understood, a strong genetic link has been identified through the application of family (8, 9) and twin’s studies (10). SLE does not follow a single locus Mendelian pattern of inheritance. And as it involves both polygenic and environmental risk factors, it is a complex trait. Complex traits are multi-factorial with both genetic and environmental contributions. Genome-wide association studies (GWAS) have been successfully used to investigate the genetic basis of a disease, and this has dramatically advanced knowledge of the genetic etiology of SLE. Our recent review summarized a total of 84 genetic loci that are implicated as SLE risk (11). Despite the advances in the genetics of SLE, it is not clear how to utilize genetic information for the prediction of SLE risk or severity.

A genetic risk score (GRS) summarizes risk-associated variations by aggregating information from multiple risk single nucleotide polymorphisms (SNPs). The approach to calculate the GRS is to simply count disease-associated alleles or weighting the summed alleles by log odds ratios (OR). Recent studies (12, 13) have proposed methods that select SNPs from GWAS by linkage disequilibrium (LD) pruning and clumping and thresholding for GRS calculation. As the number of SNPs included in a GRS increases, the distribution approaches normality, even when individual risk alleles are relatively uncommon. Therefore, a GRS can be an effective means of constructing a genome-wide risk measurement that summarizes an individual’s genetic predisposition to SLE. Moreover, as GRSs pool information from multiple SNPs, each individual SNP does not strongly influence the summary measurement. Thus, the GRS is more robust to imperfect linkage for any tag SNP and causal SNP and is less sensitive to minor allele frequencies for individual SNPs (14–17).

**Figure 1:**
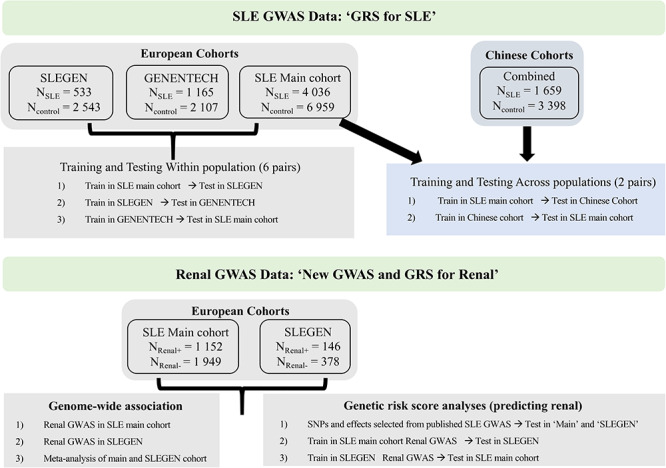
Overview study design.

Several studies (18–23) have looked at GRS for SLE; however, many relied on very few SNPs (23), had sample sizes inadequate for GRS, did not compare results across populations and were restricted to SNPs on the Immunochip. We investigated, for the first time, the performance of genome-wide SNPs for predicting SLE. As in the most recent study of LN (21), we also investigated the predictive performance of SNPs published as associated with SLE for disease severity. This study used data on three European GWAS and two Chinese GWAS (Fig. 1). We first tested whether a quantitative model, a GRS derived from SLE GWAS applying a range of methods using genome wide SNPs, was an effective way to distinguish SLE patients and controls in three independent European cohorts. Next, we classified SLE patients into two groups: SLE renal+ (patients with renal disease) and SLE renal− (patients without renal disease) and performed a case–case Renal GWAS in two independent SLE cohorts with available renal data for the identification of SLE renal susceptibility loci. We then tested whether a GRS derived from SLE GWAS or Renal GWAS was an effective way to distinguish SLE patients with or without renal disease in two independent cohorts. A GRS analysis for SLE was performed across Chinese and European data where we trained the GRS in one population and predicted in the other. The SLE risk score was elevated in those with renal disease (compared with those without), and it showed a negative correlation with the age-of-onset of the disease.

## Results

### The best GRS in SLE prediction

**Figure 2:**
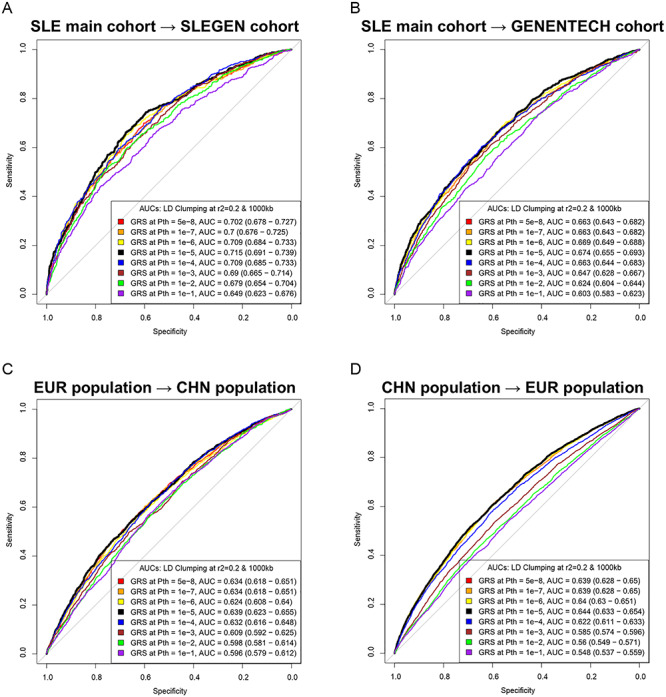
ROCs and AUCs of models in SLE prediction in European cohorts and between ancestries. GRSs for the prediction of SLE in the SLEGEN cohort (**A**) and Genentech cohort (**B**) were generated from SNPs of LD clumping, and threshold derived from the SLE main cohort. All GRSs for the training-and-validation in European cohorts were generated with two MHC tag SNPs derived from the European GWAS (See Materials and Methods). GRSs for the prediction of SLE across populations (**C**) and (**D**) were generated from SNPs of LD clumping and threshold without MHC tag SNPs. The ‘GRS at *P*_th_’ represented the GRS in the SLE prediction model, which was derived from the LD clumping at the according GWAS *P* value threshold.

**Figure 3:**
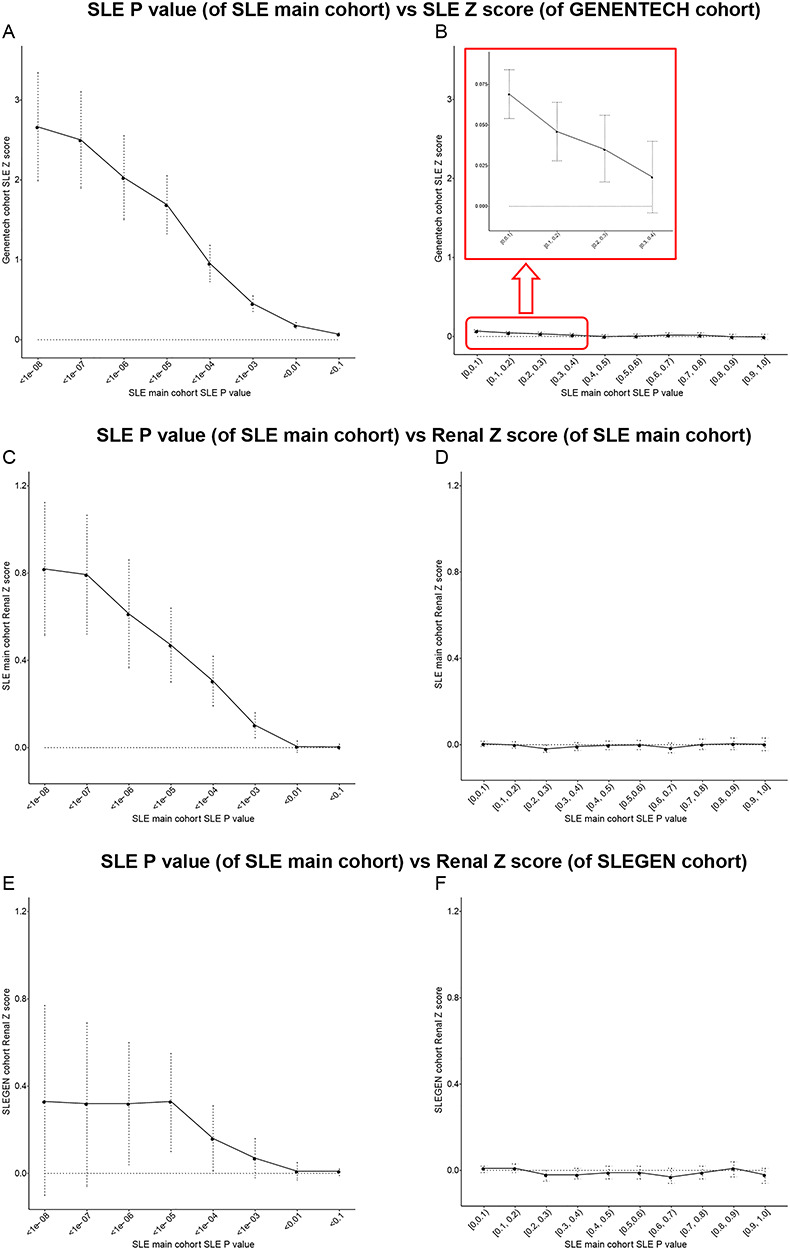
Polygenic test of SLE and renal disease. Polygenic test of SLE in genentech cohort (**A** and **B**) and polygenic test of renal disease in the SLE main cohort (**C** and **D**) and SLEGEN cohort (**E** and **F**). The SLE main cohort was used to generate a *P* value for each SNP, to stratify the SNPs into groups for the Z score calculation of SLE association or renal association.

Among the GRSs generated from LD clumping and thresholding, the predictor with the best discriminative capacity was the one derived from SNPs clumping at *P* threshold (*P*_th_) of 1e−05 with *R*^2^ < 0.2 in the SLE main cohort and tested in both the International Consortium for Systemic Lupus Erythematosus Genetics (SLEGEN) [*N*_SNPs_ = 66; area under the receiver operating characteristic (ROC) curve (AUC) = 0.72; 95% confidence interval (CI) = 0.69–0.74] and Genentech (*N*_SNPs_ = 79; AUC = 0.67; 95% CI = 0.66–0.69) cohorts (Fig. 2 and Supplementary Material, Table S1), suggesting there may be more true positive signals than the genome-wide significant ones involved in the risk of SLE. This performance was not due to the population structure as the GRS added significantly more (*P* = 2.2e−16 and 7.78e−14) to the AUC than principal components in both Genentech and SLEGEN, respectively. In fact, the predictive performance of the GRS using all pairs of training and test data was maximized using SNPs below the standard genome-wide threshold (Supplementary Material, Table S1). This evidence for polygenicity was also seen in an analysis of the association statistics (*Z* scores) in the Genentech GWAS polarized to the risk allele in the main GWAS, partitioned by their association *P* value in the main GWAS (see Materials and Methods). Here, we found evidence (Fig. 3 and Supplementary Material, Table S2) against a zero mean (*P* = 3.91e−04) for the Z scores in Genentech data for SNPs with *P* values between 0.3 and 0.2 in the main GWAS. The GRS effect was independent of a sex effect (see Supplementary Material, Table S3) with no evidence of an interaction.

We found that the GRS trained in our European (EUR) data predicted SLE in the Chinese (CHN) data well (Fig. 2C and D) with an AUC (0.64) when using the best approach for GRS in the Europeans (*R*^2^ < 0.2 for all SNP pairs and using SNPs that passed the *P* value threshold of 1e−05). The range of AUC values over all *P* value thresholds for SNP inclusion was [0.60–0.64]. The results when training in the CHN and predicting in EUR were similar: AUC = 0.64 when using the best approach for GRS in the Europeans (*R*^2^ < 0.2 for all SNP pairs and using SNPs that passed the *P* value threshold of 1e−05) and the range of AUC values over all *P* value thresholds for SNP inclusion was [0.55–0.64].

### LN GWAS within SLE cases

LN occurs in approximately half of all SLE patients, and its frequency ranges from 25 to 75% depending on the population studied (24). About one-third of European SLE patients experience renal disease (25). Until recently, one of the most common causes of death in SLE patients was kidney failure. According to the lupus severity index (LSI) using the ACR criteria developed by Bello *et al.* (26), renal involvement has the highest impact and particular strongly associated with disease severity; hence, we chose LN as a proxy of SLE severity in this study.

The imputed within case LN GWAS in the SLE main cohort, which comprised 1152 SLE patients with renal disease (LN+) and 1949 patients without renal disease (LN−), did not identify any genome-wide significant associated loci (*P* ≤ 5e−08) (Supplementary Material, Fig. S1A). Consistently, no inflation (genomic inflation factor: *λ* = 1.014) was observed in the QQ plot (Supplementary Material, Figure S1D). Similarly, none of the SNPs reached genome-wide significance in the SLEGEN cohort (27) (*λ* = 1.023) (Supplementary Material, Fig. S1B and E). In addition, no variant passed genome-wide significance in the meta-analysis of the SLE main cohort and SLEGEN cohort for Renal GWAS (*λ* = 0.9565) (Supplementary Material, Fig. S1C and F). Summary association statistics for SNPs with *P* ≤ 1e−05 are provided in Supplementary Material, Tables S4 and S5. We also did not observe any significant associations when limiting the analysis to high quality (Imputation INFO = 1) and common [minor allele frequency (MAF) $\ge$ 0.02] SNPs (see Supplementary Material, Fig. S1G and H for QQ plot).

We did, however, see evidence that SNPs with very strong evidence for association with SLE (*P* ≤ 1e−05) were associated with LN. This was evident from an analysis of the renal association statistics (Z scores) polarized to the risk allele for SLE. There was a strong evidence (Fig. 3 and Supplementary Material, Table S2, *P* = 8.72e−08) against a zero mean for the Renal Z scores for SNPs with *P* ≤ 1e−05 for SLE in the main cohort. This result was replicated in the SLEGEN study with *P* = 2.42e−03 (Fig. 3 and Supplementary Material, Table S2). The finding of renal association with SNPs showing very strong evidence for association with SLE could be exploited for the prediction of disease progression, and we explore this below.

### Genetic risk loading of SLE is significantly higher in LN+ patients

**Figure 4:**
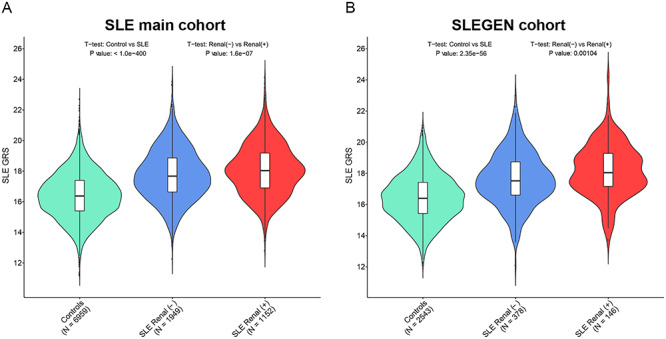
GRS over levels of disease: controls/SLE renal (−)/SLE renal (+). The violin-and-box plots show the summary GRS for each level of the disease in the SLE main cohort (**A**) and the SLEGEN cohort (**B**). The violins show the distribution of the GRS across each group. The bottom line of the box inside the violin is the first quantile, the top line is the third quantile and the box is divided at the median. Sample size (*N*) of each group is showed within brackets below the group name. Note that GRSs for SLE main cohort and SLEGEN cohort are generated by 93 non-MHC SNPs and 2 MHC tag SNPs—a total of 95 SNPs (Supplementary Material, Table S9).

While we observed that no individual SNPs were significantly associated with renal involvement in the SLE cases, we did show that there was a deviation from zero mean for renal Z scores taken from SNPs with very strong evidence for association with SLE. We checked whether a Renal GWAS derived GRS could predict renal disease; however, the performance was not good (highest AUC = 0.55 using SNPs with *P* < 0.1) and was outperformed by the SLE derived GRS (see Supplementary Material, Table S1). In view of this finding, we investigated the correlation between the SLE GRS and renal disease in all SLE cases. To accomplish this, we used the GRS derived from a list of published SLE associated SNPs (See Materials and Methods) (11) for the comparison of the SLE genetic risk burden in patients with and without renal disease. As expected, the GRS was higher in the SLE patients compared with healthy controls in both independent cohorts (Fig. 4).

A significantly higher GRS was observed in the group of patients with renal disease (LN+) compared with patients without renal disease (LN−) (Fig. 4). In the SLE main cohort, the mean (SD) of the GRS was 18.1 (1.64) for LN+ patients and 17.8 (1.65) for LN− patients (*P =* 1.60e−07); the mean (SD) for the SLEGEN cohort was 18.2 (1.66) for LN+ patients and 17.6 (1.69) for LN− patients (*P =* 0.0010). Moreover, we saw a significant increasing trend of GRS over levels of diseases: healthy control, LN− patients and LN+ patients, in the SLE main cohort and the SLEGEN cohort (Fig. 4**).**

### Genetic risk of nephritis and age-of-onset in SLE

**Figure 5:**
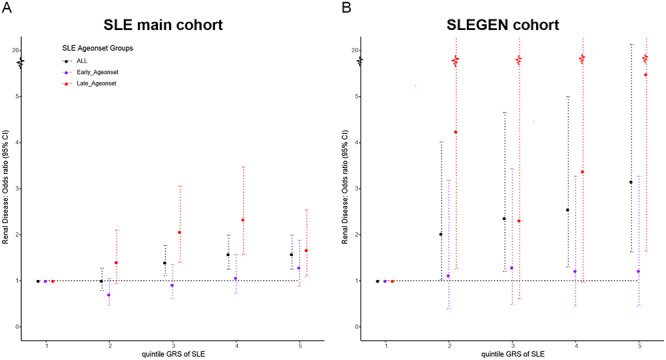
Relationship of quintiles of the GRS and risk of renal disease within SLE patients. Plots show the ORs of renal disease for the SLE main cohort (**A**) and the SLEGEN cohort (**B**), comparing each of the upper four GRS quintiles with the lowest quintile; dotted lines represent the 95% CI; horizontal black dotted lines represent OR = 1.

**Figure 6:**
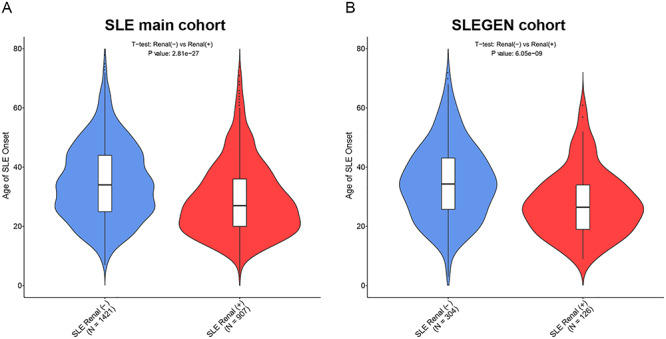
Age of SLE onset in patients of renal (−)/renal (+). The violin-and-box plots show the age of SLE onset for each level of the disease in the SLE main cohort (**A**) and the SLEGEN cohort (**B**). The violins show the distribution of the Age of SLE onset across each group. The bottom line of the box inside the violin is the first quantile, the top line is the third quantile and the box is divided at the median. Sample size (*N*) of each group is showed within brackets below the group name.

We partitioned the SLE cases into five groups according to quintiles for GRS to show the risk of renal involvement. We observed over 1.5-fold higher risk of renal disease (OR = 1.58; 95% CI = 1.25–1.99; *P =* 0.00015) between the top and bottom quintiles of GRS in the SLE main cohort (Fig. 5A). This is replicated in the SLEGEN cohort (Fig. 5B), with ORs of 3.16 (95% CI = 1.62–6.13; *P* = 0.00091). A significantly earlier age of SLE onset was observed in those with renal disease compared with those without renal disease. In the main cohort (Fig. 6A), the mean (SD) for the age of disease onset was 29 years (12) for LN+ patients and 35 years (13) for LN− patients (*P =* 2.8e−27); the means for the SLEGEN cohort (Fig. 6B) were 28 years (11) and 35 years (13) for LN+ and LN−, respectively (*P =* 6.05e−09). When testing the association of GRS with age-of-onset in the SLE main cohort, a significant correlation was present—the higher the GRS, the earlier age of SLE onset (*P =* 4.59e−12). This correlation was also detected in the SLEGEN cohort (*P =* 0.021) and the combined Chinese cohort (*P* = 1.57e−06).

**Figure 7:**
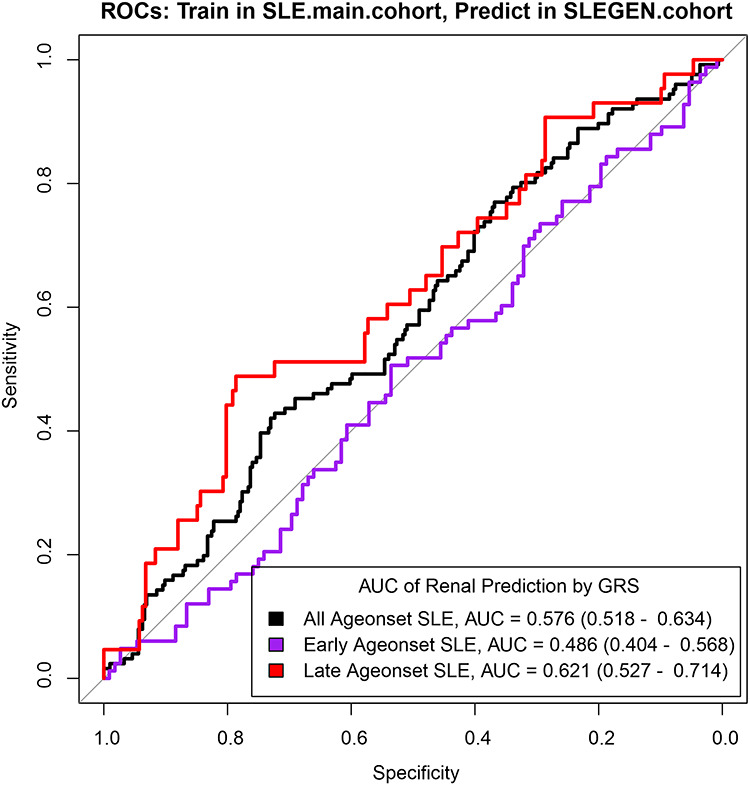
ROC curves for models predicting a diagnosis of renal disease in SLE patients using GRS, split by age-of-onset. The models were trained in the SLE main cohort and tested in the SLEGEN cohort. The plots showed the ROC curves in the prediction of renal disease in SLE patients with GRS as a predictor. The ROC curve in black was trained and tested with all SLE samples, the purple curve was trained and tested in the ‘Early age onset’ patients (≤ 30 years) and the red curve was trained and tested in the ‘Late age onset’ group. AUC, area under the ROC curve, is showed with 95% CI in brackets.

To test whether the GRS correlated with renal disease independently of age-of-onset, we partitioned SLE patients into two groups according to their age-of-onset, i.e. ‘Late age onset’ and ‘Early age onset’, and performed a two-way ANOVA test (See Materials and Methods). The GRS was shown to positively correlate with both renal disease and early age-of-onset (*P*_Renal_ = 7.64e−05 and *P*_age-of-onset_ = 1.06e−09) in the SLE main cohort, with significant association with renal disease in the SLEGEN cohort but marginal evidence for age-of-onset (*P*_Renal_ = 0.0288 and *P*_age-of-onset_ = 0.0513), whereas we found that there was no statistically significant interaction between renal and early age-of-onset in the SLE main cohort (*P*_Interaction_ = 0.795) and marginal evidence in the SLEGEN cohort (*P*_Interaction_ = 0.0511) (Supplementary Material, Fig. S2). Notably, we found that GRS was a better predictor of renal disease in the ‘Late age onset’ group (AUC = 0.62) compared with the ‘Early age onset’ group (Fig. 7). We also find that age-of-onset as a continuous trait using logistic regression is correlated with renal disease independently of the GRS (*P*_age-of-onset_ = 7.54e−23, *P*_GRS_ = 2.98e−04 in the main cohort, and *P*_age-of-onset_ = 3.68e−07, *P*_GRS_ = 3.2e−02 in the SLEGEN cohort) with no evidence of an interaction term.

Finally, we assessed the predictive ability of the partitioned SLE GRS (quintile GRS, see Materials and Methods) over the two age-of-onset groups. In the main SLE cohort, there is a clear and significant risk effect for renal involvement with increasing GRS in the ‘Late age-of-onset’ group, but no significant effect in the early onset group. We observed over 2-fold higher risk of renal disease (OR = 2.33; 95% CI = 1.57–3.47; *P =* 3.76–05) between the upper fourth quintile and the bottom quintile in the ‘Late age onset’ group in the SLE main cohort (Fig. 5A). The results were similar in the SLEGEN cohort, with the risk of renal disease between the top and bottom quintile of GRS being over five times (OR = 5.48; 95% CI = 1.65–18.3; *P* = 0.00664) (Fig. 5B and Supplementary Material, Table S6) in patients of ‘Late age onset’ but no significant differences in those with ‘Early age onset’. These results are robust to the chosen threshold in the definition of ‘Late age onset’ and ‘Early age onset’ (Supplementary Material, Table S6).

## Discussion

GRS has been showed to be predictive for several diseases including cardiovascular disease (AUC = 0.81, 95% CI = 0.81–0.81) (12), inflammatory bowel disease (AUC = 0.63, 95% CI = 0.62–0.64) (12) and breast cancer (AUC = 0.63, 95% CI = 0.63–0.65) (28). However, in many of these applications, the AUC values are dependent on the inclusion of age and sex for prediction, and therefore, the AUC due to genetics alone would have been substantially lower (29). We have shown that an SLE GRS using only SNPs has good predictive power with AUC approaching 0.7 over a range of settings when trained and tested between three European GWAS. We also used two combined Chinese studies’ data as both independent validation and a test of cross populations prediction performance. In both populations, we show that, when using GWAS data as a training set, a GRS using SNPs with associated *P* values well below genome-wide levels of significance has the best predictive performance. This, along with other studies that have reinvestigated SLE GWAS data (30), is further evidence that SLE is a polygenic disease with many risk variants as yet undiscovered, and that more powerful studies could lead to useful predictive models. While we did find that the GRS correlated with SLE independently of a sex effect (risk for females), with no evidence of interaction, the low prevalence of male SLE (approximately 10% of cases in our data) meant that we could not determine if a GRS could predict one sex better than the other. Multiple well-powered GWAS in terms of sample sizes for male cases for training and predicting would help determine the utility of sex-specific risk scores. GRSs may also have utility in the prediction of disease severity, and we find evidence for this to be so for SLE. Our data show that renal involvement is not related to specific genetic factors or particular genes but simply to genetic load of risk alleles.

Until recently, the most common cause of death in SLE patients was kidney failure. Though the frequency of death from kidney disease has decreased sharply due to better therapies (e.g*.* dialysis and kidney transplantation), kidney failure is still potentially fatal in some people with SLE and causes significant morbidity. According to the LSI using the ACR criteria developed by Bello *et al.* (26), renal involvement had the highest impact and particularly more strongly associated with disease severity; hence, we used renal involvement as a proxy of SLE severity in this study. In the SLE within-case renal GWASs, we observed no genome-wide significant signals in either the SLE main cohort or the SLEGEN cohort, or meta-analysis of these two. Both data sets had genetic variants with less stringent *P* values (*P* ≤ 1e−05) for renal association, but none of them were replicated in the other cohort. Considering the sample size of both cohorts is relatively small, we applied an online genetic power calculator (http://zzz.bwh.harvard.edu/gpc/) to calculate the power of our current sample size for the GWAS study (Supplementary Material, Table S7). We assumed that the effect sizes of SLE renal risk alleles are similar to that seen in SLE GWAS, so the OR of the risk allele would be between 1.0 and 2.0. Therefore, we calculated the power under a variety of parameters, including OR, risk allele frequency (RAF) and alpha. As showed in Supplementary Material, Table S7, we have a power of ≥0.8 to detect a genetic risk variant with an OR = 1.4 and RAF = 0.3 or an OR = 1.5 and RAF = 0.2 when alpha = 5e−08. However, if we assume that the renal associated variants are as weak as most of the SLE associated variants (OR < 1.2), then we are under powered (<0.8) to detect the true renal associations at the GWAS significant threshold of *P* = 5e−08 in the current study.

We did, however, find evidence that SNPs most associated with SLE (*P* < 1e−05) were enriched for associations with SLE renal involvement. Specifically, the renal association *P* values of the 95 SNPs (of 77 published SLE risk loci) in the SLE main cohort and the SLEGEN cohort are strongly inflated as shown in the QQ plots (Supplementary Material, Fig. S3), suggesting the cumulative genetic burden from multiple SLE risk genes with modest effect. Therefore, we then tested the hypothesis that the genetic risk loading of SLE may correlate with kidney involvement. Therefore, a GRS using published SNPs with robust evidence for association with SLE was derived for the prediction of SLE renal disease. In both European cohorts, the SLE main cohort and the SLEGEN cohort, the GRS was significantly higher in patients with renal disease than patients without. In addition, patients with a higher GRS were more likely to have renal involvement at a younger age, indicating the strong genetic background of SLE development. These findings provide more evidence to support the opinion that younger age onset lupus is generally more severe than older onset lupus as reported previously (31–33). An improvement to our study would be to use the Imputed SLE GWAS as a reference data set. This would derive a better fine-mapped set of SNPs which, as the performance of the published SNPs suggests, may have better predictive performance; however, imputed data must be converted to genotype calls for LD clumping and this loss of information reduces accuracy, and so only, very-high-quality imputed SNPs can be used, which reduces the utility. The next release of the 1Kg data will have higher coverage outside of coding regions, which is where the majority of SLE associated variants are, and should result in more accurate imputation and useful data for GRS.

Our analysis of renal disease in SLE patients has shown that, while we find no SNPs significantly associated with renal disease, the fact that SLE associated variants correlated with renal using a GRS suggests that many SLE associated variants are also risk for renal involvement albeit with likely weaker effects (ORs). We find that the GRS and age-of-onset are correlated but the GRS is associated with renal involvement independently of age-of-onset with no interaction observed. The GRS performs better for predicting renal disease in patients with late age-of-onset. We also find that a stratified GRS may be a more viable option for predicting renal disease, where we estimate significantly high relative risks for those in the tails of the GRS distribution in both of our European studies that had renal data.

A limitation of this study is that we were not able to replicate our renal results in the Chinese as renal data were not available. Renal involvement in Chinese is more common than in Europeans; the Chinese SLE patients are more heterogeneous, and those who suffer from more severe clinical manifestations and earlier age-of-onset. The use of GRS for predicting SLE severity in Chinese may not have the same utility as in Europeans where we find the stronger association in the late onset patients. Nevertheless, our results in Chinese showing a correlation between age-of-onset and SLE GRS suggest that in this population, disease severity is also driven by the load of disease-associated variants.

This is the first study to investigate accumulated genetic risk and its relationship with the susceptibility and severity of SLE with data in Chinese and European populations. We found that the higher the GRS, the younger onset of SLE in both populations. Within the European population and across the Chinese and European populations, we find that a GRS incorporating LD pruned SNPs (at *R*^2^ = 0.2) with modest (*P* < 1e−05) evidence for association with disease predicts SLE with AUC of 0.64 and above. In the European data, we see that in patients of late onset, a higher GRS means that the patients are more likely to suffer from more severe disease. In brief, age-of-onset incorporating a GRS may assist early prediction of LN in a clinical setting. Nevertheless, more clinical studies and multi-population data are needed to validate the usefulness of this application.

## Materials and Methods

### Samples source

European samples were from three previously published SLE GWAS—the SLE main cohort (34), the SLEGEN cohort (27) and the Genentech cohort (35). The SLE main cohort (34) was the biggest SLE GWAS, which consisted of 4036 SLE patients and 6959 healthy controls. A total number of 603 208 SNPs were available post-quality control (QC). The SLEGEN cohort (27) was carried out by the SLEGEN on women of European ancestry, which comprised 283 211 SNPs genotyped for 2542 controls and 533 SLE patients. The Genentech cohort (35) was performed by Genentech on North American individuals of European descent, which comprised 487 208 SNPs genotyped for 1165 cases and 2107 controls. The samples used from the three European GWAS were independent: the main GWAS publication used identity by descent (IBD) analysis in PLINK 1.9b (www.cog-genomics.org/plink/1.9/) (36) to remove individuals from Genentech with IBD > 0.125; we used these data and applied the same analysis to the SLEGEN data.

Chinese samples were from previously published GWAS from Anhui (1047 cases and 1205 controls) (37) and Hong Kong (612 cases and 2193 controls) (38, 39).

Clinical sub-phenotypes were available for the SLE main cohort and SLEGEN cohort, which were documented according to the standard American College of Rheumatology (ACR) classification criteria. Sub-groups of patients with renal disease or without renal disease were identified according to the sub-phenotype data using ACR classification. Following QC, the sample size of patients with renal disease, lupus nephritis (LN+), was 1152 and 146; while the sample size of patients without renal disease (LN-) was 1949 and 378 in the SLE main cohort and SLEGEN cohort, respectively. More details are presented in Supplementary Material, Table S8.

## Genome-wide association study

### SLE GWAS

SLE GWASs were performed in genotyped SNPs including principal components consistent with the original publications in all three independent cohorts. The two post-QC Chinese data sets, which were both typed on the Illumina 610-Quad Human beadchip, from the original studies were combined. The combined data were subjected to standard GWAS QC, and in addition, we tested for differential missing and relatedness between the two studies. Post-QC, there were 484 813 SNPs. The combined Chinese GWAS used a covariate for study. The original studies did not use principal components as covariates as the PCs clustered well due to the samples being recruited relatively locally. We conducted PCA on the combined data. See Supplementary Material, Fig. S4. for the PCA plot. The Anhui data have a single cluster. The Hong Kong (HK) data are mostly clustered to the right of the Anhui samples on PC1. There are some HK samples (40 cases and 249 controls) clustered with the Anhui samples; however, these had both cases and controls represented (7% of cases and 11% of controls).

### SLE renal GWAS within SLE cases

The SLE Renal GWASs were performed within SLE cases, i.e. genome-wide associations of patients with renal disease (SLE renal+, cases) and patients without renal disease (SLE renal−, controls) in two independent cohorts, i.e. the SLE main cohort and the SLEGEN cohort. For Renal GWASs, we pre-phased the genotyped data using the SHAPEIT algorithm (40) and then used IMPUTE2 (41) to impute to the density of the 1000 genome reference data (phase three integrated set, release 20 130 502) (42) (unpublished data). All case-control analyses were carried out using the SNPTEST algorithm (43). SNPs with imputation INFO scores of < 0.7 and MAF < 0.001 were removed. After QC, there were 21 431 070 SNPs left for further analysis. Moreover, a genome-wide association meta-analysis of the SLE main cohort and SLEGEN cohort was performed using the summary statistics derived from the two Renal GWASs. A standard threshold of *P*$\le$ 5e−08 was used to report genome-wide significance and *P* $\le$ 1e−05 was used to report suggestive associated signals.

### Polygenic analysis

We tested for non-zero standardized effect sizes (Z scores) for SLE association in the Genentech data for groups of SNPs stratified by their *P* values in the SLE main cohort. The Z scores in the Genentech data were polarized with respect to the SLE main cohort in that the effect allele was set to be the risk allele in the SLE main cohort. Under the null hypothesis, the Z scores will have zero mean, while under the alternative, the mean will be positive. SNPs were stratified by *P* value intervals of 1–0.9, 0.9–0.8, 0.8–0.7, 0.7–0.6, 0.6–0.5, 0.5–0.4, 0.4–0.3, 0.3–0.2, 0.2–0.1 and 0.1–0.00. We would expect a positive mean for SNPs with very small *P* values in the main SLE cohort as these will be enriched for true positives, while the same is not necessarily true over other *P* values ranges unless there are more widespread true associations with very weak effects. We also ran this analysis on renal association standardized effect sizes (Z scores) again polarized with respect to SLE association and stratified by SLE *P* values. In all analyses, we used an LD clumped set of SNPs with an *R*^2^ threshold of 0.1. When comparing the SLE main cohort to the Genentech cohort or the SLEGEN cohort, we limited the clumping to SNPs that overlap the GWASs.

### GRS derivation

A GRS is a quantitative trait of an individual’s inherited risk based on the cumulative impact of many genetic variants, which is calculated according to the method described by Hughes *et al.* (44).

We used two approaches to select SNPs for GRS calculation. The first approach, a weighted GRS, was derived from all published independent SLE risk SNPs (Supplementary Material, Table S9)—including 78 SLE susceptibility loci (without the X chromosome), consisting of 93 SNPs outside of the MHC region and 2 independent tag SNPs in the MHC region for two well-known HLA haplotypes in SLE, i.e. rs2187668 for HLA-DRB1*03:01 and rs9267992 for HLA-DRB1*15:01 for the European cohort and rs9271366 for HLA-DRB1/HLA-DQA1 and rs9275328 for HLA-DQB1/HLA-DQA2 for the Chinese cohort (Supplementary Material, Table S9). The risk allele for each SNP is derived from its original publication, which is summarized in a recent review (11), and the effect size used in the GRS was generated from each GWAS used as a training set. Each GRS for four SLE cohorts (27, 34, 37–39) was generated using R version 3.4.3.

The second approach—LD clumping and thresholding—was used to build 32 GRSs. Clumping and thresholding scores were built using a *P* value and LD-driven clumping threshold in PLINK version 1.90b (www.cog-genomics.org/plink/1.9/) (36). In brief, the algorithm forms clumps around SNPs with associated *P* values less than a provided threshold (index SNPs). Each clump contains all SNPs within a specified window of the index SNP which are also in LD with the index SNP as determined by a provided pairwise correlation threshold (*r*^2^) in the training data. The algorithm loops through all index SNPs, beginning with the smallest *P* value and only allowing each SNP to appear in one clump. The final output should contain the most significant disease-associated SNP for each LD-based clump across the genome. We found that including the MHC region in the clumping algorithm performed worse that a GRS excluding the MHC, which could be due to overfitting in the training set and different LD patterns across data; so we included tag SNPs for the well-known MHC risk haplotypes. When performing LD clumping, we firstly removed the X-chromosome and the MHC extended region (24–36 MB) and kept all other autosomal SNPs. Then, we included the MHC region by using two tag SNPs for two well-known HLA haplotypes in SLE (Supplementary Material, Table S9). The MHC tagSNPs were only added to the GRS in the cross validation within the European population. A GRS was built using the genotypes for the index SNPs weighted by the estimated effect sizes (β). Specifically, when training the GRS in the SLE main cohort and testing in the SLEGEN cohort, we performed a GWAS on the genotyped SNPs in the SLE main cohort and generated 32 lists of clumped SNPs over a set of *P* values (*-clump-p1*: 0.1, 0.01, 1e−03, 1e−04, 1e−05, 1e−06, 1e−07 and 5e−08), *r*^2^ (*-clump-r2:* 0.2 and 0,5) and clumping radius (*-clump-kb*: 250 and 1000). The 32 lists of SNPs were then used to generate 32 GRSs by summing across all variants weighted by their respective effect size for samples in the SLEGEN cohort. We performed this analysis using all three cohorts in European population with one data set as training and the other as a test set, generating six training-and-testing pairs. We also performed a cross population analysis between European and Chinese populations. There were 270 268 SNPs overlapping the European and Chinese data.

### ROC curves for model evaluation

The GRS with the best discriminative capacity was determined based on the maximal AUC with SLE or RENAL as the outcome and the candidate GRS as the predictor. AUC CIs were calculated using the ‘*pROC*’ package within R, and the difference between the ROC curves was determined with the ‘*roc.test’* function, which used a non-parametric approach, as described by De Long *et al.* (45). To assess the degree to which the age of SLE onset contributes to the prediction of renal involvement within SLE cases, we generated ROCs as above with the GRS and compared with ROC curves with SLE age onset as a single predictor and the ROC with both GRS and age onset as predictor(s).

### Partitioning the genetic risk of renal disease

Since a continuous score is difficult to interpret on an individual level when a physician needs to explain the results of the GRS to a patient, we partitioned SLE patients into quintile according to genetic dosage (SLE GRS). We used a chi-square test to study the association of the partitioned GRS and renal risk. The ORs of renal risk were then calculated compared to the reference group—the first quintile GRS group.

To test whether the GRS correlated with renal disease independently of age-of-onset, we partitioned SLE patients into two groups according to their age-of-onset, with a cut-off at the age of 30—patients with age above 30 were defined as ‘Late age onset’ and others as ‘Early age onset’. A two-way ANOVA test was then performed with the function ‘*aov*’ in R, with aov(GRS ~ age group * renal group). All statistical analyses were conducted using R version 3.4.3 software (https://www.r-project.org/).

## Supplementary Material

Supplementary_material_Tables_ddaa030

Supplementary_material_Figures_ddaa030

## Abbreviations

GRS genetic risk scores; GWAS, genome-wide association studies; LD, linkage disequilibrium; LN, lupus nephritis; OR, odds ratio; ROC, receiver operating characteristic; SLE, systemic lupus erythematosus; SNP, single nucleotide polymorphisms
