# Supplementary material for: Genome-wide assessment of genetic risk for systemic lupus erythematosus and disease severity
Source: Hum Mol Genet. 2020 Feb 20;29(10):1745–56. doi: 10.1093/hmg/ddaa030 (PMC7322569; doi:10.1093/hmg/ddaa030)
Supplement: Supplementary_material_Figures_ddaa030 [file supplementary_material_figures_ddaa030.docx]

| **A** | **B** | **C** |
| --- | --- | --- |
| **** | | |
| **D** | **E** | **F** |
| **** | | |
| **G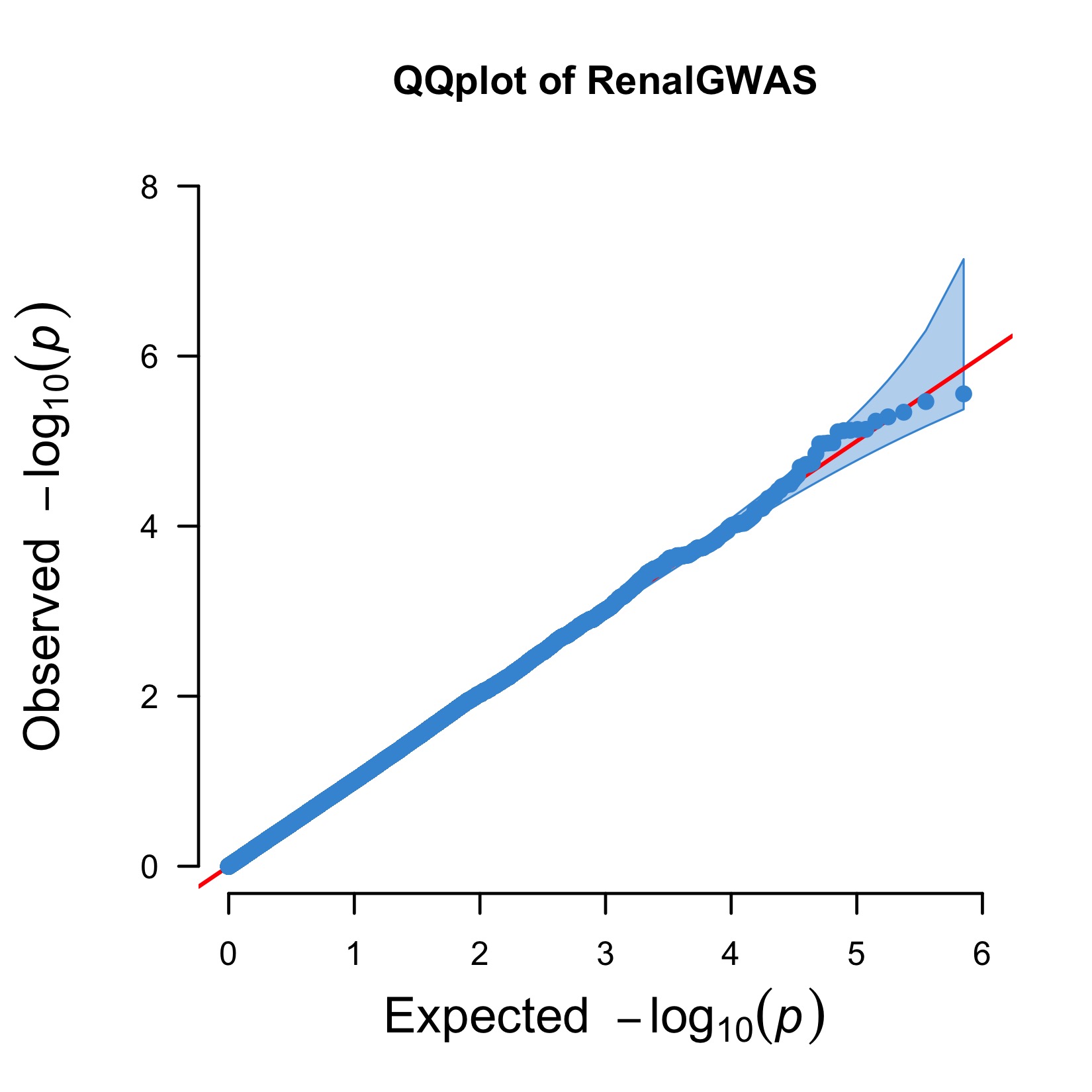** | **H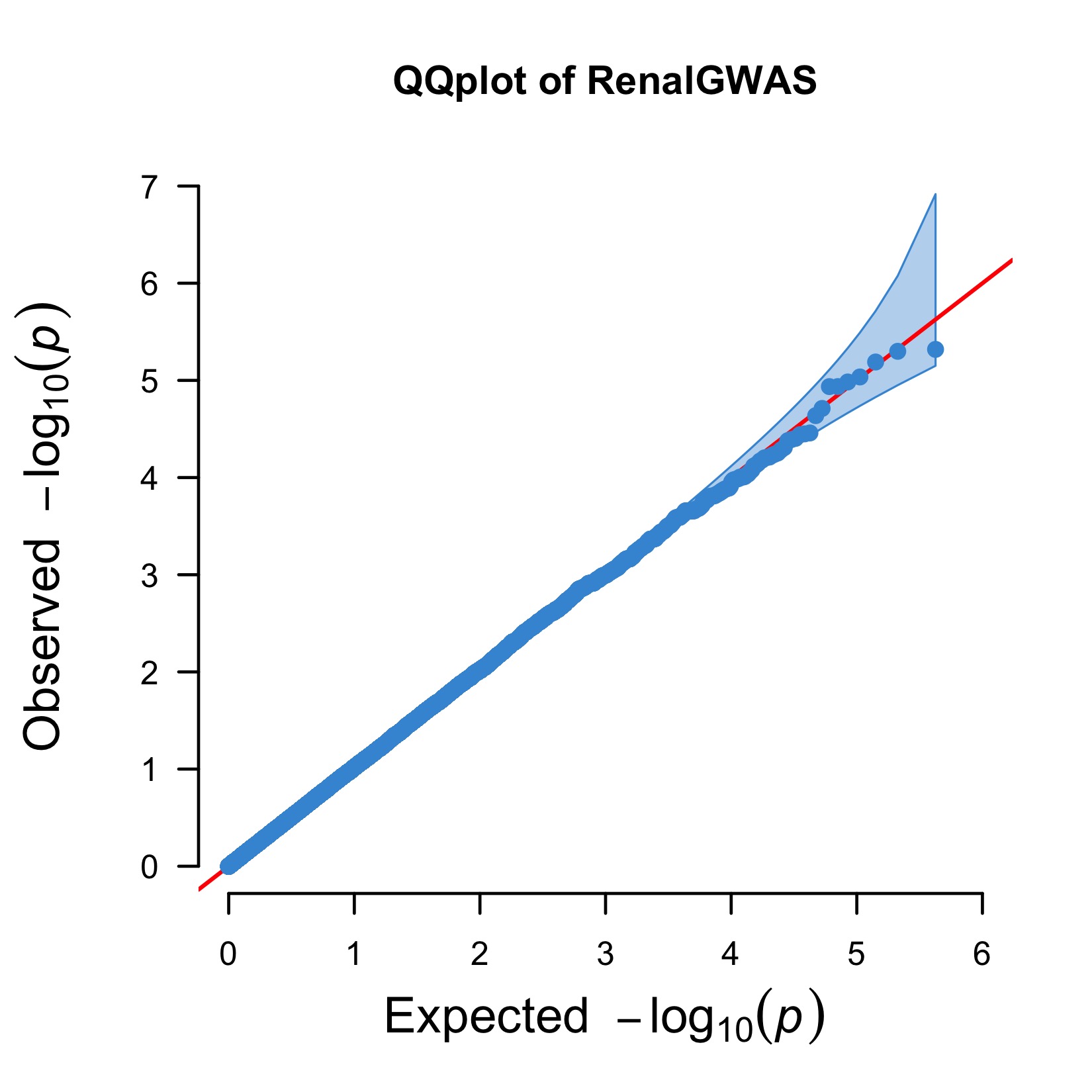** |  |

Figure S1. Genome-wide scans of LN associated variants.

(Upper) Manhattan plots showing the -log10-transformed p values (y axis) against physical genomic position (x axis) for each SNP in the SLE main cohort (**A**), the SLEGEN cohort (**B**), and the meta-analysis of these two cohorts (**C**). The red horizontal line represents the threshold for genome-wide significance (P ≤ 5e-08) and the blue horizontal line represents the threshold for suggestive significance (P ≤ 1e-05). (Middle) Quantile-quantile plots (QQ-plot) showing the observed distribution of -log10-transformed p values (y axis) by the expected distribution (x axis) under the null hypothesis of no association (diagonal line) for the SLE main cohort (genomic inﬂation factor, λ = 1.014) (**D**), the SLEGEN cohort (λ = 1.023) (**E**), and the meta-analysis of these two cohorts (λ = 0.9565) (**F**). (Lower) QQ-plots showing subset of SNPs with high quality for the SLE main cohort (**G**) and the SLEGEN cohort (**H**).

|  |
| --- |

Figure S2. Relationship of GRS and age onset in Renal disease.

The age of SLE onset ≤ 30 years was defined as “Early onset” and > 30 years was defined as “Late onset”. For each age onset and renal group, the GRS was plotted with mean and 95% CI for the SLE main cohort.

| **A SLE main cohort** | **B SLEGEN cohort** |
| --- | --- |
|  |  |

Figure S3. Quantile-quantile plots of Renal association results

QQ plots showed the observed distribution of -log10-transformed p values (y axis) by the expected distribution (x axis) under the null hypothesis of no association (diagonal line) for the SLE main cohort (**A**) and the SLEGEN cohort (**B**). The *P* values for the QQ plots were derived from Renal association test of the 95 SNPs (**Table S8**) which used for the GRS calculation.


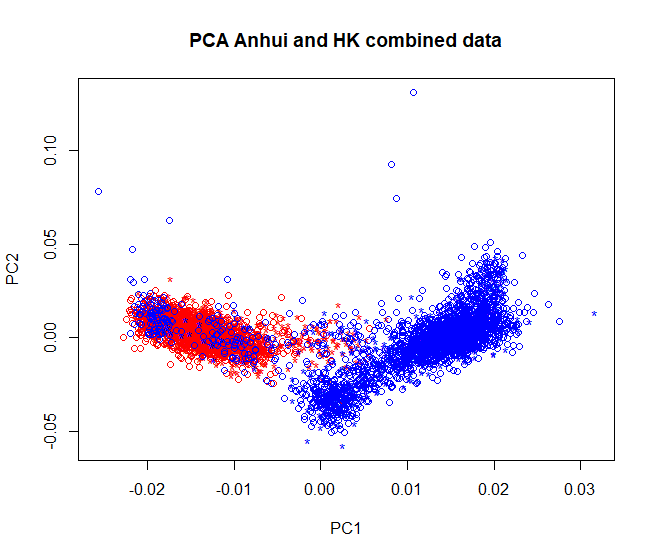


Figure S4. PCA plot of Chinese combined data

Blue: HKcohort; Red: Anhui cohort.

Circle ( $^{\circ}$ ) represents healthy controls; start ( $*$ ) represents SLE cases.
